# Mapping the orbital structure of impurity bound states in a superconductor

**DOI:** 10.1038/ncomms15175

**Published:** 2017-05-08

**Authors:** Deung-Jang Choi, Carmen Rubio-Verdú, Joeri de Bruijckere, Miguel M. Ugeda, Nicolás Lorente, Jose Ignacio Pascual

**Affiliations:** 1 CIC nanoGUNE, 20018 San Sebastián-Donostia, Spain; 2 Kavli Institute of Nanoscience, Delft University of Technology, 2628 CJ Delft, The Netherlands; 3 Ikerbasque, Basque Foundation for Science, 48013 Bilbao, Spain; 4 Centro de Física de Materiales CFM-CSIC, 20018 San Sebastián-Donostia, Spain; 5 Donostia International Physics Center (DIPC), 20018 San Sebastián-Donostia, Spain

## Abstract

A magnetic atom inside a superconductor locally distorts superconductivity. It scatters Cooper pairs as a potential with broken time-reversal symmetry, leading to localized bound states with subgap excitation energies, named Shiba states. Most conventional approaches regarding Shiba states treat magnetic impurities as point scatterers with isotropic exchange interaction. Here, we show that the number and the shape of Shiba states are correlated to the spin-polarized atomic orbitals of the impurity, hybridized with the superconductor. Using scanning tunnelling spectroscopy, we spatially map the five Shiba excitations found on subsurface chromium atoms in Pb(111), resolving their particle and hole components. While particle components resemble *d* orbitals of embedded Cr atoms, hole components differ strongly from them. Density functional theory simulations correlate the orbital shapes to the magnetic ground state of the atom, and identify scattering channels and interactions, all valuable tools for designing atomic-scale superconducting devices.

## Abstract (web summary)

The complex internal structure of magnetic impurities, which locally distort superconductivity, is usually neglected. Here, Choi *et al*. report that the number and shape of Shiba states are correlated to the spin-polarized atomic orbitals of the impurity, valuable to design atomic-scale superconducting devices.

Yu-Shiba-Rusinov (Shiba) states^1^,^2^,^3^ are identified in scanning tunnelling spectra as pairs of intra-gap resonances symmetrically positioned around zero-bias^4^,^5^,^6^,^7^,^8^,^9^. Each resonance corresponds to the injection of an electron or hole into the bound state^8^, thus representing the particle or hole components of the quasiparticle (QP) wavefunction. Since Shiba excitations lie inside the superconducting gap, their lifetime considerably exceed that of other QPs. This anticipates that Shiba peaks behave as a robust probe of scattering phenomena in superconductors, revealing intrinsic properties such as the distribution of the order parameter^5^, the QP band structure^10^, the effect of dimensionality^9^ or Andreev tunnelling processes^8^. Owing to their long lifetime, Shiba excitations exhibit narrow lineshapes, which enables the study of magnetic phenomena in impurities with high energy resolution, such as magnetic anisotropy^11^,^12^ and magnetic coupling^6^,^13^.

Theoretical models using classical spins with isotropic exchange fields have predicted multiple Shiba bound states, labelled by an angular momentum quantum number *l* (ref. 3). In fact, magnetic transition metal (TM) atoms in a superconductor show a varying number of Shiba bound states depending on the nature of both element and superconductor^5^,^6^,^8^,^9^,^14^,^15^. However, the exchange fields of atomic scatterers are not isotropic. Due to the orbital character of the scattering channels, the spin can only be exchange scattered by spin-polarized atomic states, which for 3*d*-TM atoms are those defined by the angular momentum *l*=2. The symmetry reduction by crystal fields, and hybridization with the host atomic lattice lift the degeneracy of the *l*=2 subshell. In this scenario, Shiba multiplets are predicted to appear, thus reflecting the occupation level of the atomic shell^16^. The resolution of their orbital character would render them as the ideal probe for identifying the magnetic ground state of a single impurity in a superconductor. Furthermore, the spatial extension of Shiba states also provides precise access to the coupling between impurities^6^ and to basic properties of the superconducting host^5^,^9^,^15^,^17^.

Here we explore possible orbital components in Shiba states. We compare the QP local density of states (LDOS) of chromium atoms deposited on Pb(111), mapped with a low-temperature scanning tunnelling microscope (STM), with atomistic simulations based on density functional theory (DFT). We find that the atoms are embedded under the first Pb layer and show five intra-gap states due to QP scattering with the five *d* orbitals of Cr. The spectral maps of the Shiba states are very localized on the impurity and show a characteristic particle-hole asymmetry. Based on theoretical maps of particle and hole components we interpret their orbital origin and obtain hints on the interaction strength for each channel.

## Results

### Multiple Shiba states of Cr atoms

**Figure 1:**
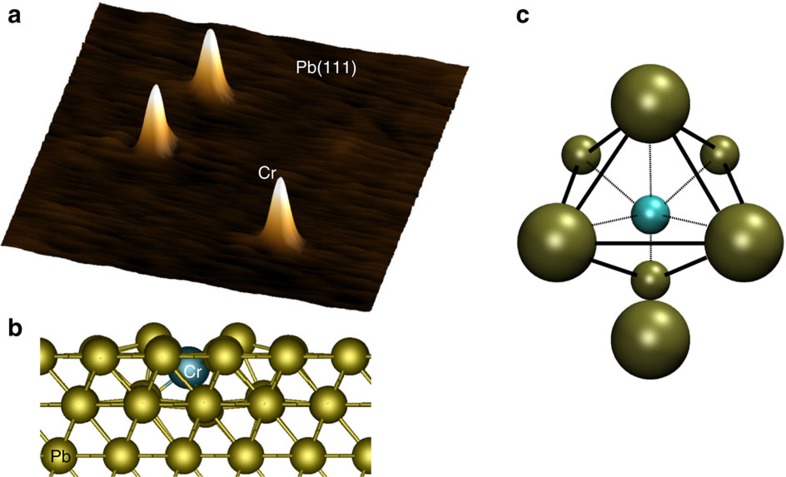
Chromium atoms in Pb(111). (**a**) Constant-current STM image of three Cr atoms in the Pb(111) surface (*V*=4 mV, *I*=10 pA, 12 × 12 nm^2^). The atom apparent height is 50 *pm* for these imaging conditions. (**b**) Result of a full DFT relaxation showing that the most stable position of a Cr atom (blue) is at a subsurface site (Pb atoms in yellow). (**c**) Top view of the local atomic surroundings of the embedded Cr atom, composed by seven Pb atoms all at a distance of ∼3±0.2 Å. The four largest Pb atoms are surface atoms. The relaxed Cr atom lies in a pseudo-FCC position, displaced towards two Pb atoms, which are slightly lifted above the surface plane. The resulting cluster resembles a distorted octahedron with an extra atom at one vertex.

We deposited Cr atoms on a Pb(111) film grown on SiC(0001) (thickness ≳100 nm) at 15 K. The atoms appear in STM images (measured at a base temperature of 1.2 K) as protrusions with a small apparent height of ∼50 pm (Fig. 1a). DFT calculations reveal that the most stable configuration corresponds to the Cr atom underneath the uppermost Pb layer, as sketched in Fig. 1b,c (Methods section and Supplementary Note 2). The barrier for reaching this state is as low as 21 meV, which is smaller than, for example, the surface lateral diffusion barriers (Supplementary Table 1). Since the Cr atoms arrive hot to the Pb(111) surface, they can easily access the embedded site before they thermalize to the substrate temperature. The simulated STM topography agrees with the small experimental height of the STM images.

**Figure 2:**
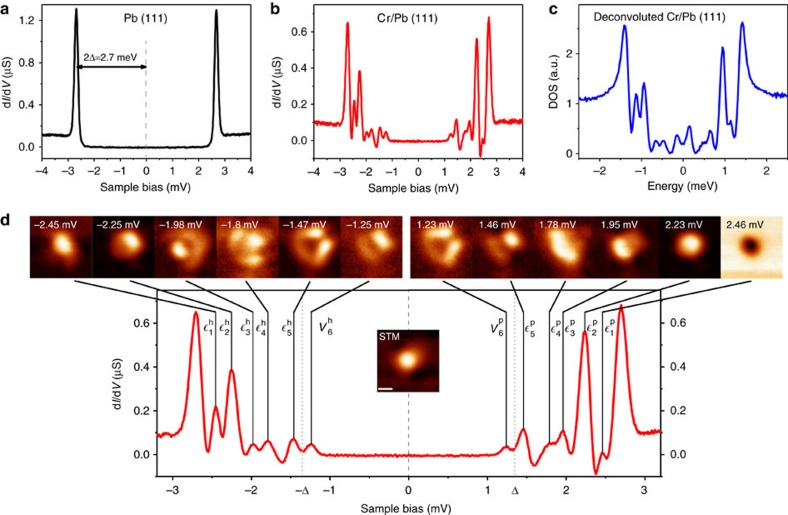
Spectrum and Shiba maps of Cr atoms. Differential conductance spectra measured with a superconducting (Pb-covered^7^,^24^) tip (**a**) on the bare Pb(111) surface and (**b**) on the Cr adatom, where intra-gap states are easily recognized. (**c**) DOS of the Cr impurity obtained by deconvoluting the tip contribution in **a** from spectrum **b**, as described in Supplementary Note 5. Five positive- and five negative-bias states are observed inside the superconducting gap, at opposed bias-polarity values, in agreement with five pairs of Shiba excitations with different intensities. Most of the differences in peak intensity are due to their inhomogeneous spatial distribution. (**d**) d*I*/d*V* maps over the Cr impurity at the energy of the intra-gap states in the spectrum (**b**) (expanded here for clarity). The maps represent the amplitude of each spectral peak obtained from a 52 × 52 matrix of d*I*/d*V* spectra acquired in an area of 2.2 nm × 2.2 nm over the chromium atom (all with set point *V*=4 mV and *I*=0.35 nA of the corresponding STM image shown in the inset). Intra-gap peaks are labelled according to their position and hole(h)/particle(p) character. Peaks 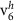/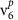 correspond, respectively, to injection of holes/electrons into states 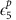/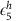, which are thermally populated by particles/holes due to their proximity to the Fermi level. Therefore, states 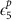 and 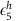 are imaged twice. Scale bar, 0.5 nm.

The QP LDOS associated to subsurface Cr impurities was obtained from differential tunnelling conductance (d*I*/d*V*) spectra using superconducting Pb-terminated tips to increase the energy resolution beyond the thermal limit^7^. Figure 2a shows a typical d*I*/d*V* spectrum acquired on bare Pb(111) that exhibits a doubled superconducting (SC) gap of Pb with sharp coherence peaks at ±2Δ=±2.7 meV (Methods section). The d*I*/d*V* curve taken on a Cr atom in Fig. 2b reveals a rich spectral structure inside the superconducting gap, with six intra-gap peaks at each polarity (that is, six peak pairs), in addition to the original Pb coherence peaks. To obtain the QP LDOS, we deconvoluted the experimental d*I*/d*V* spectrum on chromium (Fig. 2b) using the superconducting DOS of a Pb-tip, estimated from Fig. 2a (as described in Supplementary Note 5). The deconvoluted spectrum, Fig. 2c, shows just five energy-symmetric pairs of peaks, labelled as 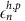 (*n*=1, 2, 3, 4, 5) in Fig. 2d. We attribute these pairs of peaks to five Shiba states with excitation energies 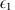
=±1.1 meV, 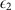
=±0.9 meV, 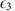
=±0.62 meV, 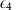
=±0.45 meV and 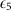
=±0.125 meV. The additional pair of peaks, denoted 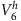 and 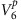 in the d*I*/d*V* spectrum of Fig. 2d, appears at bias voltages ±(Δ−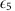)/*e* and, thus, corresponds to a thermal replica of the Shiba pair 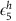/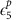 (ref. 8). Shiba multiplets arise naturally as different angular momentum components in isotropic exchange fields. However, the resolution of a fivefold Shiba multiplet achieved here enables us to reinterpret their origin in terms of atomic properties of the impurity^11^,^16^.

### Spatial shape of Shiba states

To probe the orbital origin of the Shiba bound states, we map their intensity in a small region around the Cr impurity. Figure 2d shows d*I*/d*V* maps obtained for the same Cr atom at the voltages of all twelve peaks found inside the SC gap (Methods section). In contrast to the featureless protrusion in topography (inset of Fig. 2d), the conductance maps reveal various features around the Cr impurity. Each Shiba state appears with a different shape, which is smoother for the first two, and shows specially marked intra-atomic features for states 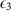 and 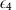, and 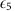. Interestingly, the maps show a clear dependence with polarity, signalling a particle-hole asymmetry in the wavefunction of the corresponding Shiba states. This is particularly clear for states 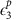 and 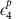, and 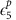, which are markedly different than their corresponding hole states. Such spatial asymmetry is also observed in the thermal replicas at 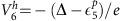 and 
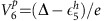
, which show the shapes of their mirror states 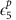 and 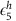, respectively. The different shape of particle and hole components of every state explains their different peak amplitude in point spectra like in Fig. 2b. In fact, d*I*/d*V* spectra averaged over the whole extension around the Cr impurity levels their intensity at both polarities (Supplementary Fig. 4).

To find out the origin of the number and shape of the Shiba states, we simulated with DFT (Methods section) the scattering channels of the embedded Cr impurity and modelled their effect in producing Shiba bound states. The channels are very sensitive to atomic-scale details of the Cr atom and its environment. We employed the most stable Cr site in the subsurface plane described in Fig. 1b, and found that this site produced results compatible with the experiment.

**Figure 3:**
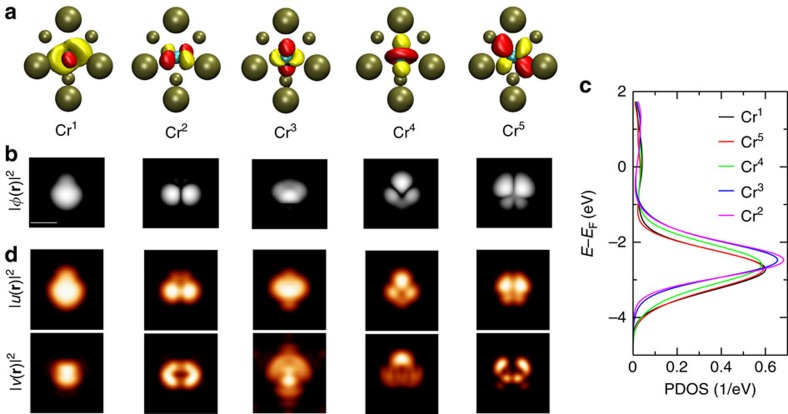
DFT results of scattering states and Shiba states. (**a**) Top view of wavefunction amplitude isosurfaces for the five electronic states of the cluster shown in Fig. 1c. These five states are the only ones with relevant weight from the original *d*-manifold of the Cr atom, and their shape resembles the Cr *d*-electrons modified by the symmetry of the embedded site, as can be seen in the top view of their squared amplitude modulus in **b**, scale bar 0.5 nm. (**c**) Spin-polarized projected density of states (PDOS) of the full electronic structure of the Pb(111)+Cr slab (Fig. 1b) on the five cluster orbitals depicted in **a**. The cluster states are considerably broadened due to the strong coupling with the complete Pb system, and spin-polarized: only one spin component is shown here, the other component shows only PDOS well above the Fermi level. (**d**) Modulus squared of the Bogoliubov QP coefficients, that is, 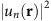 and 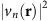, on the Pb(111) surface, calculated for the five cluster states of **a**.

The identification of Cr-derived scattering channels is difficult; the embedded atom does not lie in a fully symmetric position inside the Pb FCC crystal, while it induces a significant displacement on its first Pb neighbours. Consequently, the Cr *d* orbitals are strongly hybridized and mixed with Pb *sp* bands. To facilitate their identification in the band's continuum, we calculated first the single-electron energy levels of the cluster formed by the Cr atom and the 7 first neighbours with apparent interaction (shown in Fig. 1c). We found that, at least in this reduced ensemble, there are five non-degenerate singly occupied states with clear *d* character centred in the Cr atom. Figure 3a plots isosurfaces of wavefunction amplitude for the five eigenstates inside the atomic cluster. Their shape partly resembles some of the original symmetries of *d* orbitals. This implies that these states will mostly exchange-scatter *l*=2 host electrons^18^. However, the degeneracy of the *d* multiplet is lifted and the *m*_*l*_ orbitals are mixed by the anisotropic Pb crystal field. In the full Pb system, these states appear as broad resonances around −2.6 eV (Fig. 3c shows the DOS of the Pb slab projected on the five cluster states of Fig. 3a) due to the mixture with *sp* bands of the lead film, but their spin polarization remains very strong. We deem that the intra-gap bound states are created by exchange scattering of host electrons into these five one-electron states.

### Simulation of Shiba state images

The spatial shapes of the five Cr-states of the cluster, projected on the surface (Fig. 3b), show already some level of agreement with the experimental Shiba maps. For example, the maps of peaks 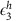, 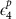 or 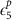 resemble the shapes of states Cr^3^, Cr^4^ or Cr^5^, respectively. This validates the subsurface atomic configuration obtained by DFT because the mixture of Cr-*d* orbitals is very sensitive to fine details of the atomic crystal field environment. To correlate orbital states with subgap impurity bound states, we computed the corresponding Shiba states by considering that each of them scatters with conduction electrons via a one-electron potential. The effect of an impurity on the total Hamiltonian can be divided in a potential scattering term (*K*(**r**), no spin degrees of freedom) and a spin contribution^3^, such that


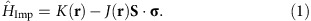


where *J*(**r**) is the exchange-coupling between the impurity spin S and the 1/2 spin of conduction electrons **σ**. In a first approximation, we consider that the five cluster eigenstates of Fig. 3a also diagonalize these contributions, giving rise to five spin-polarized scattering channels. Since their degeneracy is lifted, they have a different exchange interaction *J*_*μ*_ with the host and produce a different bound state. They are then responsible for the appearance of five positive-energy (particle) and five negative-energy (hole) Shiba states in the QP LDOS, symmetrically aligned with respect to the Fermi energy^16^. The DFT results indicate that for this system the potential scattering terms *K*_*μ*_ in equation (1) are very small. In the absence of a potential scattering term, a large degree of particle-hole symmetry in the weight and amplitude of the Shiba states is expected^5^,^19^. This is indeed the case after adding up all differential conductance spectra measured over the surface region around the Cr impurity, as commented above and shown in Supplementary Fig. 4.

The spatial distribution of Shiba amplitudes can be depicted by the squared modulus of the Bogoliubov QP coefficients, 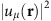 and 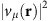, representing the LDOS of their particle and hole components, respectively^20^. Figure 3d plots the quantities 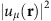 and 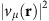 over the embedded Cr impurity, obtained as described in Supplementary Note 3. The results reproduce the different shape of particle and hole states from the experiments, especially for those resonances with marked intra-atomic features. We find that the particle components 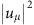 resemble the shape of Cr *d* states in Fig. 3c. This is a result of the extended Fermi surface of lead, which provides a continuous set of scattering wave-vectors that partly reconstruct the shape of the scattering state. However, the hole components 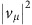 strongly deviate for the orbital shapes. This is due to the dominating spin contribution to the potential: QP scattering with hole components produces a phase reversal for wavevectors of the Fermi surface, resulting in a clear distortion of the shape of scattering channels.

## Discussion

We can recognize several features from the simulated particle and hole components of Bogoliubov QPs (Fig. 3d) in the experimental conductance maps of Shiba states (Fig. 2d). In particular, the particle components 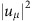 of orbitals Cr^3^ and Cr^5^ resemble the positive bias maps of Shiba states 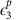 and 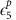, respectively, whereas their 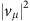 fits well with the corresponding negative-bias maps, 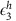 and 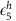. This situation is indicative of an exchange constant *J*_*μ*_ smaller than the superconducting paring energy, so that tunnelling into the particle component of the bound superconducting pair requires extra positive energy. An unexpected particle-hole reversal is found for the Shiba bound state 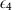, where the calculated particle component (and orbital shape) clearly matches the d*I*/d*V* map at negative bias. When *J*_*μ*_ is larger than the pairing energy, the bound superconducting pair breaks and the new ground state is a Bogoliubov QP, while the scattering spin channel becomes screened (*s*=0). A fingerprint of this new ground state is an energy reversal of its particle and hole excitations^21^, while their crossing through zero energy signals the transition to this new ground state^7^,^12^,^21^. Thus, the resemblance of 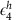 to 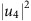 denotes that the corresponding orbital experiences a larger interaction with the Pb bands (that is, a larger hybridization, compatible with our DFT results, Supplementary Note 4), becoming fully screened and not contributing to the total atomic spin.

The sharp maps of Shiba states change shape rapidly between peaks, in barely <200 μeV, a much greater accuracy than any other method of orbital imaging one-electron states, and in spite of the large degree of hybridization of the buried atom. This is ensured by the superconducting gap in the QP spectrum, which keeps Shiba states with long excitation lifetimes and decoupled from other QPs (except indirectly via thermal effects and/or via Andreev processes^8^). Moreover, Shiba states are fairly unperturbed by the presence of nearby impurities. Our measurements, shown in Supplementary Fig. 5, reveal that two impurities separated by only 4 surface lattice parameters (1.4 nm) display largely undisturbed Shiba conductance maps, what is attributed to the large localization of Shiba states in three dimensions^3^,^9^. This suggests that coupling impurity-induced bound states together into extended magnetic structures^6^,^22^,^23^ requires impurity separations well in the sub-nm range. In these structures, identification of the characteristic orbital symmetries of impurity bound states is thus a unique fingerprint to unveil their (channel-specific) magnetic ground state, their magnetic coupling to other nearby impurities, and to follow the formation of extended Shiba bands.

## Methods

### STM measurements

The experiments were conducted in a commercial SPECS GmbH Low-Temperature (1.2 K) STM, under Ultra-High Vacuum conditions. Pb(111) films (thickness ≳100 nm) on SiC(0001) substrates appear as crystalline grains with diameters >300 nm, which show bulk-like superconductivity. To increase the energy resolution, the STM tip was repeatedly embedded in the Pb film until a superconducting tip was obtained. The full superconducting state of the tip was proved by performing STS spectra on bare Pb regions and showing that the two sharp coherence peaks appear at ±2Δ, where Δ=1.35 meV, as in Fig. 2a. Contrary to single-crystal measurements^24^ the spectra on bare Pb films never showed the characteristic double gap structure, probably due to the small film thickness compared to the Pb coherence length scale^25^, which washes out the anisotropy of the Fermi Surface^26^. Cr atoms were deposited on the Pb(111) surface at a surface temperature of ∼15 K. The shown spectra were obtained using a lock-in amplifier, with modulation *V*_rms_=10 μV at 938.5 Hz. The Shiba conductance maps were obtained from a matrix of 52 × 52 d*I*/d*V*(*V*) spectra measured in a region of 4.8 nm^2^ over the Cr impurity. The corresponding topography images do not show any of the features observed in the Shiba maps (see inset of Fig. 2d). Analysis of STM and STS data were performed with the WS × M^27^ and SpectraFox^28^ (http://www.spectrafox.com) software packages.

### Theoretical details

Standard calculations using DFT were performed to reproduce the total energy and the electronic structure of different configurations of a Cr atom on Pb(111) (Supplementary Note 1). The calculations unequivocally predict a subsurface configuration under the surface bridge site, with considerable distortion of the surface Pb layer. A minimal Pb–Cr cluster is used to determine the local electronic structure near the Cr atom. Using the wavefunctions of this cluster, the Bogoliubov-de Gennes equations are approximated, in the presence of an exchange field, obtaining the equivalent of Rusinov's equations using generalized scattering channels and a non-free-electron normal metal. The equations are further approximated to yield the spatial distribution of the Shiba states, as detailed in the Supplementary Information.

### Data availability

The data that support the findings of this study are available from the corresponding authors upon request.

## Additional information

**How to cite this article:** Choi, D.-J. *et al*. Mapping the orbital structure of impurity bound states in a superconductor. *Nat. Commun.* **8,** 15175 doi: 10.1038/ncomms15175 (2017).

**Publisher's note**: Springer Nature remains neutral with regard to jurisdictional claims in published maps and institutional affiliations.

## Supplementary Material

Supplementary Information Supplementary Notes and Supplementary References

## References

[b1] Yu L. Bound state in superconductors with paramagnetic impurities. Acta Phys. Sin. 21, 75–91 (1965).

[b2] Shiba H. Classical spins in superconductors. Prog. Theor. Phys. 40, 435–451 (1968).

[b3] Rusinov A. I. On the theory of gapless superconductivity in alloys containing paramagnetic impurities. Sov. J. Exp. Theor. Phys. 29, 1101–1106 (1969).

[b4] Yazdani A., Jones B. A., Lutz C. P., Crommie M. F. & Eigler D. M. Probing the local effects of magnetic impurities on superconductivity. Science 275, 1767–1770 (1997). 9065395 10.1126/science.275.5307.1767

[b5] Hudson E. W. . Interplay of magnetism and high-Tc superconductivity at individual Ni impurity atoms in Bi_2_Sr_2_CaCu_2_O_8_+*δ*. Nature 411, 920–924 (2001). 11418850 10.1038/35082019

[b6] Ji S.-H. . High-resolution scanning tunneling spectroscopy of magnetic impurity induced bound states in the superconducting gap of pb thin films. Phys. Rev. Lett. 100, 226801 (2008). 18643441 10.1103/PhysRevLett.100.226801

[b7] Franke K. J., Schulze G. & Pascual J. I. Competition of superconducting phenomena and kondo screening at the nanoscale. Science 332, 940–944 (2011). 21596987 10.1126/science.1202204

[b8] Ruby M. . Tunneling processes into localized subgap states in superconductors. Phys. Rev. Lett. 115, 087001 (2015). 26340200 10.1103/PhysRevLett.115.087001

[b9] Gerbold C. M. . Coherent long-range magnetic bound states in a superconductor. Nat. Phys. 11, 1013–1016 (2015).

[b10] McElroy K. . Relating atomic-scale electronic phenomena to wave-like quasiparticle states in superconducting Bi_2_Sr_2_CaCu_2_O_8_+delta. Nature 422, 592–596 (2003). 12686994 10.1038/nature01496

[b11] Žitko R., Bodensiek O. & Pruschke T. Effects of magnetic anisotropy on the subgap excitations induced by quantum impurities in a superconducting host. Phys. Rev. B 83, 054512 (2011).

[b12] Hatter N., Heinrich B. W., Ruby M., Pascual J. I. & Franke K. J. Magnetic anisotropy in shiba bound states across a quantum phase transition. Nat. Commun. 6, 8988 (2015). 26603561 10.1038/ncomms9988 PMC4674822

[b13] Yao N. Y. . Phase diagram and excitations of a Shiba molecule. Phys. Rev. B 90, 241108 (2014).

[b14] Pan S. . Imaging the effects of individual zinc impurity atoms on superconductivity in Bi_2_Sr_2_CaCu_2_O_8_+delta. Nature 403, 746–750 (2000). 10693798 10.1038/35001534

[b15] Ruby M. . Orbital picture of yu-shiba-rusinov multiplets. Phys. Rev. Lett. 117, 186801 (2016). 27835014 10.1103/PhysRevLett.117.186801

[b16] Moca C., Demler E., Jankó B. & Zaránd G. Spin-resolved spectra of Shiba multiplets from Mn impurities in MgB2. Phys. Rev. B 77, 174516 (2008).

[b17] Randeria M. T., Feldman B. E., Drozdov I. K. & Yazdani A. Scanning Josephson spectroscopy on the atomic scale. Phys. Rev. B 93, 161115 (2016).

[b18] Schrieffer J. R. The Kondo effect—the link between magnetic and nonmagnetic impurities in metals? J. Appl. Phys. 38, 1143–1150 (1967).

[b19] Flatté M. E. Quasiparticle resonant states as a probe of short-range electronic structure and andreév coherence. Phys. Rev. B 61, R14920–R14923 (2000).

[b20] Fujita K. . Bogoliubov angle and visualization of particle-hole mixture in superconductors. Phys. Rev. B 78, 054510 (2008).

[b21] Salkola M. I., Balatsky A. V. & Schrieffer J. R. Spectral properties of quasiparticle excitations induced by magnetic moments in superconductors. Phys. Rev. B 55, 12648–12661 (1997).

[b22] Nadj-Perge S. . Observation of majorana fermions in ferromagnetic atomic chains on a superconductor. Science 346, 602–607 (2014). 25278507 10.1126/science.1259327

[b23] Ruby M. . End states and subgap structure in proximity-coupled chains of magnetic adatoms. Phys. Rev. Lett. 115, 197204 (2015). 26588411 10.1103/PhysRevLett.115.197204

[b24] Ruby M., Heinrich B. W., Pascual J. I. & Franke K. J. Experimental demonstration of a two-band superconducting state for lead using scanning tunneling spectroscopy. Phys. Rev. Lett. 114, 157001 (2015). 25933331 10.1103/PhysRevLett.114.157001

[b25] Rochlin G. Determination of the anisotropy of the energy gap in superconducting pb by superconductive tunneling. Phys. Rev. 153, 513–532 (1967).

[b26] Norman S. Far-infrared absorption spectra of thick superconducting films. Phys. Rev. 167, 393–407 (1968).

[b27] Horcas I. . WSXM: A Software for Scanning Probe Microscopy and a Tool for Nanotechnology. Rev. Sci. Instrum. 78, 013705 (2007). 17503926 10.1063/1.2432410

[b28] Ruby M. SpectraFox: a free open-source data management and analysis tool for scanning probe microscopy and spectroscopy. SoftwareX 5, 31–36 (2016).

