# Supplementary material for: Mapping the orbital structure of impurity bound states in a superconductor
Source: Nat Commun. 2017 May 8;8:15175. doi: 10.1038/ncomms15175 (PMC5424157; doi:10.1038/ncomms15175)
Supplement: Supplementary Information — Supplementary Notes and Supplementary References [file ncomms15175-s1.pdf]

SUPPLEMENTARY NOTE 1 - THE ADSORPTION OF Cr ATOMS ON Pb(111)

Chromium atoms were directly evaporated into the sample stage of our SPECS JT-STM, hosting the Pb(111) film at $\sim$ 15K . The STM topography of individual Cr atoms shows a distinctive elongated shape, signaling a 2-fold symmetry of the adsorption site (Supplementary Fig. 1a). The longitudinal axis of the elongated shapes points towards the three main crystallographic axis of the Pb(111) surface (see Supplementary Fig. 1c and 1d). The spectroscopic features are identical for the three differently adsorbed Cr atoms, just with spatial shapes rotated 120°. The height of Cr atom is relatively low, typically amounting $\sim$ 50 pm, as for the atom shown in Fig. S1a (Supplementary Fig. 1b). Both, the low height and elongated shapes along crystallographic direction agree with our DFT findings shown in the main text, and described next.

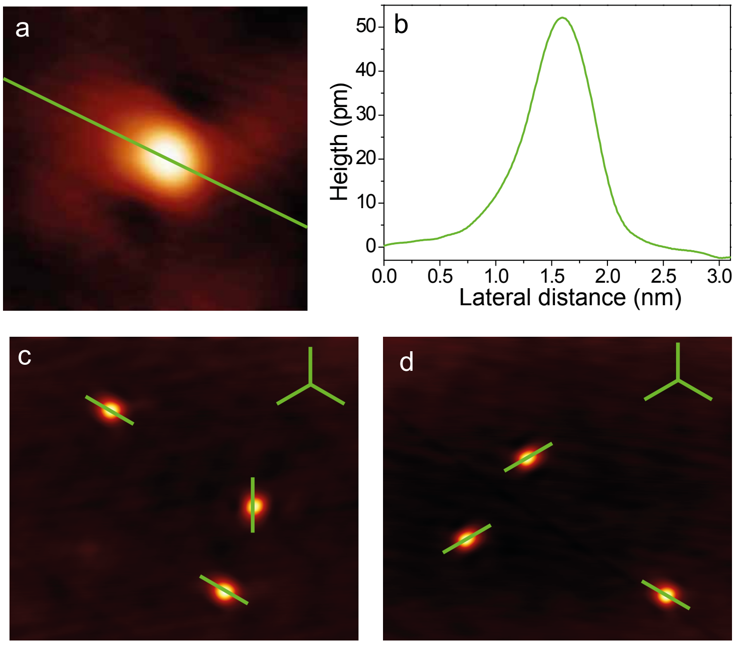

Supplementary Figure 1: Topographic images of single Cr atoms on Pb(111). (a) STM image of a Cr atom on the Pb(111) surface. They appear as elongated protrusions ($V = 4$ mV, $I = 10$ pA, size: 13×13 nm^2). (b) Height profile of Cr atom in (a). The height of the atom is $\sim 50\,pm$. (c) and (d) shows STM images of a 13×13 nm^2 area obtained with the same parameters. The elongation of Cr atoms points towards three directions separated by 120°.

SUPPLEMENTARY NOTE 2 - DENSITY FUNCTIONAL THEORY CALCULATIONS

Density Functional Theory calculations were performed using the VASP [9] code. We simulated the Cr/Pb(111) system with a slab of 5 Pb layers and a 5×5 Pb(111) surface unit cell. We used the PBE exchange-and-correlation functional [13] with the Dudarev *et al* [3] correction for the intra-atomic correlation of the Cr d-manifold, $U_{\text{eff}} = 4$ eV, together with the PAW method [2] and a cutoff energy of 250 eV. The k-point sampling was $3 \times 3 \times 1$. Forces were relaxed until they were smaller than 0.02 eV/Å.

	top	bridge	FCC	HCP
Surface	−0.812	−1.209	−1.263	−1.268
Subsurface	-	−1.483	−1.411	−1.121

Supplementary Table I: Adsorption energies of a Cr atom at different positions. Adsorption energies in eV of a Cr atom at different surface (atop the first Pb (111) layer), or subsurface (between the first and second layers) sites. The subsurface bridge site corresponds to an adsorption configuration where the Cr atom is below the first layer but between the FCC and bridge sites.

We find a lattice parameter for the Pb crystal of 5.02 Å. This is 0.07 Å larger than the experimental one as expected from the generalized-gradient approximation PBE. We computed the adsorption energy (energy gain with respect to having a Cr atom in the gas phase and a perfect surface) of a Cr atom at several high-symmetry sites on the Pb(111) surface and subsurface. The results are shown in Supplementary Table I. Subsurface adsorption is more favourable for both FCC-hollow sites and bridge sites, but less favourable for HCP-hollow sites due to the presence of the second-layer Pb atom. Next, we computed the potential barrier height for a Cr atom to reach a sub-surface FCC hollow site, and found that this was only 21 meV. Hence, (hot) Cr atoms deposited onto the Pb(111) surface can easily cross this barrier and relax into a subsurface site before dissipating their thermal energy.

However, Cr atoms show lower symmetry in STM images (two-fold, as seen in Supplementary Fig. 1). Allowing

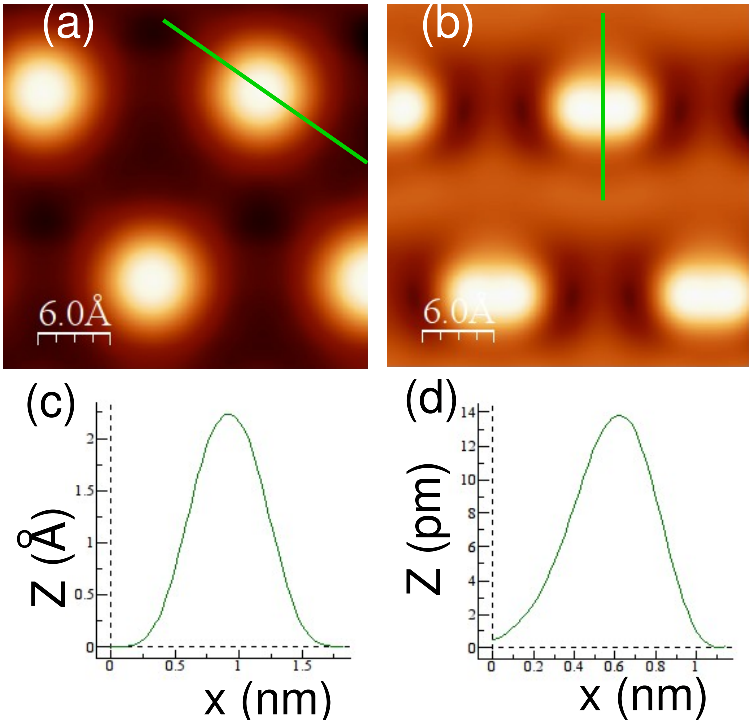

Supplementary Figure 2: Simulation of the differently adsorbed Cr atoms. (a) Simulation of the STM image of a Cr atom adsorbed above the Pb(111) surface. (b) Simulation of a Cr atom adsorbed underneath the first surface layer, for the configuration of minimum energy discussed in this article. The two-fold symmetry of the experimental image (elongated shape as shown in Fig. 2d of the manuscript and Supplementary Fig. 1) is reproduced by the subsurface atom configuration. (c) Line profile of the on-surface atom, obtained along the green line of the image of (a). (d) Line profile of the subsurface atom, along the green line of (b). The corrugation drops substantially for the subsurface atom.

full relaxation of a Cr atom at an initial subsurface FCC site, we find that it slightly drifts towards the bridge site, finding its lower-energy configuration (E_{adsorp}=-1.483 eV) at an intermediate point between subsurface bridge and FCC hollow sites. As shown in Figure 1 of the main text, in this configuration the two closer bridge atoms on top are slightly displaced upwards, and the Cr atom sits in the center of an (slightly deformed) octahedron of Pb atoms approximately 3.2 Å away. A seventh Pb atom of the surface also lies within this distance, and the interaction of the Cr impurity with the Pb crystal can be reduced to these 7 neighbouring Pb atoms. This configuration exhibits a mirror plane, perpendicular to the (111) surface, in good agreement with the symmetry of the measured topography. Indeed, the simulated constant-current STM images give excellent agreement with the experimental images only for the subsurface configuration. The constant-current images are taken as contours of constant density of states at the tip position following the Tersoff-Hamman theory. Figure S2 also shows simulated constant current images for the on-surface configuration, panel (a), and for the subsurface one, panel (b). The 2-fold symmetry is only obtained for the subsurface configuration, Supplementary Fig. 2(b). Furthermore, the corrugation of the constant-current image drops well below one Angstrom only for the subsurface case, in good agreement with the experimental corrugation of 50 pm. Figures S2 (c) and (d) show the large change of corrugation for a Cr atom adsorbed on the surface, panel (c), and subsurface, panel (d).

Figure 3c in the main text shows the (spin-polarized) density of states projected on the d-manifold of the minimal cluster of Fig. 1, for a Cr atom embedded in Pb (111) as described above. Since this cluster is a component of the Cr-Pb system, the electronic hybridization between the cluster states with a large d-component (orbitals from 1 to 5 in Fig. 3) is large, leading to broad occupied peaks in the PDOS. The corresponding d-derived states with opposite spin polarization are unoccupied and lie above the vacuum level in the present calculation, showing that the system is strongly spin polarized. Indeed, the above magnetic moments corroborate this picture, Table II. The crystal field around the Cr atom splits the d-manifold. We see a tendency to degenerate two pairs of states as one would expect from an octahedral symmetry, however the calculations show that the symmetry is lower due to the partial reconstruction of the surface and the inclusion of a seventh atom in the minimal cluster. As a consequence all degeneracies are lifted and five different states are recovered. These Cr-Pb states give rise to five distinct Shiba states.

	top	bridge	FCC	HCP
Surface	5.1	4.9	4.9	4.9
Subsurface	-	4.1	4.0	4.1

Supplementary Table II: Atomic magnetic moments in μ_B of the adsorbed Cr atom. On the surface, the Cr atom largely keeps its nominal S=5/2 spin, but in between the first and second Pb layers, the spin drops to S=2.

Table II shows the magnetic moments of the Cr atoms in the above adsorption sites. As the coordination with Pb atoms increases the magnetic moment decreases due to the hybridization of the Cr d-manifold with the Pb bands. On the surface, the spin of the Cr atom is 5/2. The preferred subsurface adsorption leads to a reduction of the Cr spin to S=2.

SUPPLEMENTARY NOTE 3 - EVALUATION OF SHIBA STATE IMAGES

The theory of Shiba states is well covered in the literature [11, 16–18]. Rusinov includes all ingredients in his excellent article on superconductivity near a paramagnetic impurity [16]. Here, we use his methodology, extending it to the scattering states of the impurity as done by Moca and co-workers [11]. As explained by Balatsky *et al* [1, 5] we can approximate the STM image by $|u^{\mu}_{\uparrow}(\boldsymbol{r})|^2$ for positive bias and by $|v^{\mu}_{\downarrow}(\boldsymbol{r})|^2$ for negative bias. Here, u and v are the quasiparticle amplitudes that enter the Bogoliubov-de Gennes equations [16]. The superscript μ denotes the scattering channel. As remarked by Moca and co-workers [11], we can use the Kondo approximation written in terms of scattering channels to treat the scattering of one electron off the paramagentic impurity. We use the definition of eigenchannels given by Paulsson and Brandbyge [12]. We can see that the eigenchannels will diagonalize the hybridization function [8], given by:

$$\Gamma_{m,m'}(\omega) = \sum_{n\boldsymbol{k}} V_{m,n\boldsymbol{k}} V_{n\boldsymbol{k},m'} \delta(\omega - \epsilon_{n\boldsymbol{k}}). \tag{1}$$

Here $V_{m,n\boldsymbol{k}}$ is the one-electron hybridization between the impurity's orbitals, m and the substrate's Bloch wave functions, given by $n, \boldsymbol{k}$, at energy $\epsilon_{n\boldsymbol{k}}$. By virtue of the Schrieffer-Wolf transformation, the eigenchannels also

diagonalize the potential and Kondo scattering interactions:

$$\hat{H}_{imp} = \sum_{\mu,\alpha} K^\mu \hat{\psi}^\dagger_{\mu,\alpha}(\boldsymbol{r})\hat{\psi}_{\mu,\alpha}(\boldsymbol{r}) - \frac{1}{2}\sum_{\mu,\alpha,\beta} J^\mu \hat{\psi}^\dagger_{\mu,\alpha}(\boldsymbol{r})\boldsymbol{\sigma}_{\alpha,\beta}\cdot \boldsymbol{S}^\mu \hat{\psi}_{\mu,\beta}(\boldsymbol{r}). \tag{2}$$

In this expression, K and J are the matrix elements of the scattering potential and the exchange-coupling term, α, β are spin-$\frac{1}{2}$ indices. We explicitly obtain the scattering potential (K and J) by noting that there is a one electron potential, $V_{d,nk}$ coupling Co (d) and substrate (nk) states. If we choose d to be the set eigenchannel states μ that diagonalize Γ, then $V_{d,nk} = V^\mu_{nk}$. It is straightforward to build a spin-flip scattering potential from a one-electron one following the Schrieffer-Wolff transformation. In order to simplify the expression we assume that K and J are diagonal in band index, which they are not in general [11]. Hence, the above expression takes into account that the scattering eigenchannels given by the field operators $\hat{\psi}_{\mu,\alpha}$ diagonalize the interactions with respect to the scattering indices μ given by the impurity's d-manifold. The channel spin S^μ is either 0 or 1/2. Since we are dealing with an atom, the intra-atomic exchange will be very large and the different spin channels will compose when we consider effective expressions of the coupling in the classical spin approximation of Rusinov [16]. We will write then the total spin of the atom, $\boldsymbol{S}$, instead of the channel one. Finally, the K and J matrix elements are given by the expressions:

$$\begin{aligned} K^\mu &= -\frac{\Gamma_\mu (U^\mu + 2E^\mu)}{4\pi\rho_0 E^\mu (E^\mu + U^\mu)} \\ J^\mu &= -\frac{\Gamma_\mu U^\mu}{4\pi\rho_0 E^\mu (E^\mu + U^\mu)} \end{aligned} \tag{3}$$

where Γ^μ are the eigenvalues of Eq. (1), E^μ are the energy of the orbital d-levels at the origin of the scattering channels as given by the above $V_{d,nk} = V^\mu_{nk}$, U^μ is the intra-atomic Coulomb repulsion for the same orbital and ρ_0 is the density of states at the Fermi energy of the normal phase. These expressions neglect the small corrections due to the superconducting gap and are the Kondo limit of the full Schrieffer-Wolf transformation.

The Shiba states can now be found by looking at solutions inside the superconducting gap Δ. The in-gap scattering prevents spin-flips so that we can just take the non-spin-flip components of Eq. (2). We adopt the Nambu notation following Shiba [17] such that the impurity Hamiltonian acts on a Nambu wavefunction, $\Psi(\boldsymbol{r})$, as:

$$\hat{H}_{imp}\Psi(\boldsymbol{r}) = \sum_{\mu,\alpha,\beta} (K^\mu \rho_z \delta_{\alpha,\beta} - \frac{1}{2} J^\mu S \sigma_z \rho_z)\psi_{\mu,\beta}(\boldsymbol{r})\langle \psi_{\mu,\alpha}|\Psi\rangle. \tag{4}$$

In this equation, tensorial products are not written and σ_z and ρ_z are the z Pauli matrices for the spin and spatial sectors. The full Hamiltonian is divided in two, following Rusinov [16]. The superconductor is treated in the BCS model in the Nambu space. Hence, the Shiba states are solutions of:

$$[\hat{H}_{BCS} + \hat{H}_{imp}]\Psi(\boldsymbol{r}) = \epsilon \Psi(\boldsymbol{r}) \tag{5}$$

where ϵ is the energy of the Shiba state. We can solve the above equation using the BCS resolvent, $G_{BCS}(\boldsymbol{r}-\boldsymbol{r}')$, obtaining

$$\Psi(\boldsymbol{r}) = \int G_{BCS}(\boldsymbol{r}-\boldsymbol{r}')\hat{H}_{imp}\Psi(\boldsymbol{r}')d^3r'. \tag{6}$$

The resolvent is given in terms of the normal-metal Bloch functions $\phi_{n\boldsymbol{k}\alpha}(\boldsymbol{r})$ by

$$G_{BCS}(\boldsymbol{r}-\boldsymbol{r}') = \sum_{n\boldsymbol{k}\alpha} \frac{\phi_{n\boldsymbol{k}\alpha}(\boldsymbol{r})\phi^*_{n\boldsymbol{k}\alpha}(\boldsymbol{r}')}{\epsilon^2 - \xi^2_{n\boldsymbol{k}} - \Delta^2_n}[\epsilon + \xi_{n\boldsymbol{k}}\rho_z + \Delta_n \sigma_y \rho_y]. \tag{7}$$

Including the above equation in Eq. (6) together with Eq. (4), we obtain the equivalent of Rusinov's equations using generalized scattering channels and a non-free-electron normal metal:

$$\begin{aligned} u^\mu_\uparrow(\boldsymbol{r}) &= \sum_{n,\boldsymbol{k},\alpha} \frac{\phi_{n\boldsymbol{k}\alpha}(\boldsymbol{r})}{\epsilon^2 - \xi^2_{n\boldsymbol{k}} - \Delta^2_n}[(\epsilon + \xi_{n\boldsymbol{k}})(K^\mu - \frac{1}{2}J^\mu S)\langle \phi_{n\boldsymbol{k}\alpha}|\psi_{\mu,\uparrow}\rangle\langle\psi_{\mu,\uparrow}|u^\mu_\uparrow\rangle \\ &+ \Delta_n (K^\mu + \frac{1}{2}J^\mu S)\langle \phi_{n\boldsymbol{k}\alpha}|\psi_{\mu,\downarrow}\rangle\langle\psi_{\mu,\downarrow}|v^\mu_\downarrow\rangle] \end{aligned} \tag{8}$$

and for the occupied part:

$$v^{\mu}_{\downarrow}(\boldsymbol{r}) \;=\; -\sum_{n,\boldsymbol{k},\alpha} \frac{\phi_{n\boldsymbol{k}\alpha}(\boldsymbol{r})}{\epsilon^2-\xi^2_{n\boldsymbol{k}}-\Delta^2_n}[(\epsilon-\xi_{n\boldsymbol{k}})(K^{\mu}+\frac{1}{2}J^{\mu}S)\langle\phi_{n\boldsymbol{k}\alpha}|\psi_{\mu,\downarrow}\rangle\langle\psi_{\mu,\downarrow}|v^{\mu}_{\downarrow}\rangle$$
$$+\;\Delta_n(K^{\mu}-\frac{1}{2}J^{\mu}S)\langle\phi_{n\boldsymbol{k}\alpha}|\psi_{\mu,\uparrow}\rangle\langle\psi_{\mu,\uparrow}|u^{\mu}_{\uparrow}\rangle]. \tag{9}$$

In order to evaluate the modulus square of the above two expressions that give rise to the computed Shiba images, Fig. 3 of the main text, we perform the following approximations. We assume we can replace the Bloch functions by simple plane waves. This reduces the computational effort to performing Fourier transforms over the first Brillouin zone, $\psi_{\mu}(\boldsymbol{k})=\langle\phi_{n\boldsymbol{k}\alpha}|\psi_{\mu}\rangle$. Furthermore, we assume that the scattering channels, $\psi_{\mu,\downarrow}$, are independent of the spin as done in a restricted Hartree-Fock approximation. In this case, the above equations are simplified to

$$u^{\mu}_{\uparrow}(\boldsymbol{r}) \;=\; \frac{1}{\sqrt{vol}}\sum_{n,\boldsymbol{k}} \frac{e^{i\boldsymbol{k}\cdot\boldsymbol{r}}}{\epsilon^2-\xi^2_{n\boldsymbol{k}}-\Delta^2_n}\psi_{\mu}(\boldsymbol{k})[(K^{\mu}-\frac{1}{2}J^{\mu}S)(\epsilon+\xi_{n\boldsymbol{k}})\langle\psi_{\mu}|u^{\mu}_{\uparrow}\rangle$$
$$+\;\Delta_n(K^{\mu}+\frac{1}{2}J^{\mu}S)\langle\psi_{\mu}|v^{\mu}_{\downarrow}\rangle] \tag{10}$$

and for the hole part:

$$v^{\mu}_{\downarrow}(\boldsymbol{r}) \;=\; -\frac{1}{\sqrt{vol}}\sum_{n,\boldsymbol{k}} \frac{e^{i\boldsymbol{k}\cdot\boldsymbol{r}}}{\epsilon^2-\xi^2_{n\boldsymbol{k}}-\Delta^2_n}\psi_{\mu}(\boldsymbol{k})[(\epsilon-\xi_{n\boldsymbol{k}})(K^{\mu}+\frac{1}{2}J^{\mu}S)\langle\psi_{\mu}|v^{\mu}_{\downarrow}\rangle$$
$$+\;\Delta_n(K^{\mu}-\frac{1}{2}J^{\mu}S)\langle\psi_{\mu}|u^{\mu}_{\uparrow}\rangle]. \tag{11}$$

Despite the obvious simplification, these equations are still difficult to solve. In order to sketch the shape of the resulting Shiba states, we have focused on the spatial components of $u^{\mu}_{\uparrow}(\boldsymbol{r})$ and $v^{\mu}_{\downarrow}(\boldsymbol{r})$. We assume that the overlaps of the scattering functions with the quasiparticles coefficients are constant during the iterations process, and we do not solve these equations self-consistently. To estimate them, we use that a single eigenchannel enters the quasiparticle functions, and then:

$$u^{\mu}_{\uparrow}(\boldsymbol{r})=\langle\boldsymbol{r}|\psi_{\mu}\rangle\langle\psi_{\mu}|u^{\mu}_{\uparrow}\rangle \tag{12}$$

and from here we obtain that

$$\frac{u^{\mu}_{\uparrow}(0)}{v^{\mu}_{\downarrow}(0)}=\frac{\langle\psi_{\mu}|u^{\mu}_{\uparrow}\rangle}{\langle\psi_{\mu}|v^{\mu}_{\downarrow}\rangle}.$$

From the usual Rusinov's equations, Ref. [16], we obtain that this ratio is approximately 0.3 for typical coupling values. According to our DFT calculations the potential scattering is much smaller than the Kondo one and we can approximate the states by

$$u^{\mu}_{\uparrow}(\boldsymbol{r})\propto\sum_{n,\boldsymbol{k}} \frac{e^{i\boldsymbol{k}\cdot\boldsymbol{r}}}{\epsilon^2-\xi^2_{n\boldsymbol{k}}-\Delta^2_n}\psi_{\mu}(\boldsymbol{k})[0.3(\epsilon+\xi_{n\boldsymbol{k}})+\Delta_n] \tag{13}$$

and for the hole part:

$$v^{\mu}_{\downarrow}(\boldsymbol{r})\propto\sum_{n,\boldsymbol{k}} \frac{e^{i\boldsymbol{k}\cdot\boldsymbol{r}}}{\epsilon^2-\xi^2_{n\boldsymbol{k}}-\Delta^2_n}\psi_{\mu}(\boldsymbol{k})[\epsilon-\xi_{n\boldsymbol{k}}-0.3\Delta_n]. \tag{14}$$

We clearly see in these equations that the quasiparticle images are made from the Fourier components of the scattering wave functions. We take as the scattering wave functions the wavefunctions of each orbital depicted in Fig. 3 of the main text, as they correctly reproduce the impurity's states in the minimal cluster around the impurity. We observe that BCS factor multiplying the Fourier components of the scattering wave functions, $1/(\epsilon^2-\xi^2_{n\boldsymbol{k}}-\Delta^2_n)$, is basically a Dirac δ function centered about the Fermi energy. Hence, it acts as a filter, selecting the Fourier components of wavevectors $\boldsymbol{k}$ of the Fermi surface.

The spatial dependence comes from the contribution of the different Fourier components, $\psi_\mu(\boldsymbol{k})$ evaluated at the Fermi sphere. The extended two Fermi surfaces of Pb assure that almost all $\boldsymbol{k}$ components are well represented in the summations. The Fourier components of the scattering wavefunctions are further weighed by a factor proportional to

$$0.3(\epsilon + \xi_{n\boldsymbol{k}}) + \Delta_n \tag{15}$$

for the empty-state quasiparticle wavefunction and

$$\epsilon - \xi_{n\boldsymbol{k}} - 0.3\Delta_n \tag{16}$$

for the occupied one.

We can easily observe that the coefficient of the hole component $v^\mu_\downarrow$, Eq. (16), changes sign at the value $\xi_{n\boldsymbol{k}} = \epsilon - 0.3\Delta_n$, which is a positive number, smaller than Δ_n. This means that, as the $\boldsymbol{k}$ approaches the Fermi wavevector $\boldsymbol{k}_F$, the Pb band $\xi_{n\boldsymbol{k}}$ approaches the value at which the factor (16) changes sign, and the components $\psi_\mu(\boldsymbol{k})$ undergo a phase-shift in the Fourier summation. However, for the particle component $u^\mu_\uparrow$, the change of sign of the factor in Eq. (15) takes place at $\xi_{n\boldsymbol{k}} = -(\epsilon + \Delta_n/0.3)$, which is beyond the values weighed by the BCS factor in the summation and, thus, all $\psi_\mu(\boldsymbol{k})$ components contribute constructively with roughly the same coefficient.

The consequences are that the empty states, $u^\mu_\uparrow$, are basically proportional to the sum of $\psi_\mu(\boldsymbol{k})e^{i\boldsymbol{k}\cdot\boldsymbol{r}}$ over the Fermi surface because there is no sign change of the coefficients for the allowed range of values. For Pb, the two Fermi surfaces expand a quite complete subset of the first Brillouin zone, leading to empty Shiba states $u^\mu_\uparrow$ maintaining the same symmetries as the scattering states, see Figure 3d of the main text. On the contrary, the filled states, $v^\mu_\downarrow$, suffer a change of sign in the summation of Fourier components. The corresponding phase shifts cause a considerably distortion of the final symmetries. For simplicity, the maps of $|u^\mu_\uparrow|^2$ and $|v^\mu_\downarrow|^2$ in Fig. 3d are constructed employing a simplified double-band Fermi surface covering most of the first Brillouin zone. We expect that Fermi surfaces with pockets and large anisotropy in k-space will induce projected shapes of Shiba states very distorted from the originating impurity states. In fact, in Refs. [10, 15] Shiba states were mapped as extended oscillations, what was attributed to highly anisotropic Fermi surfaces, with k-components focusing electrons only along certain crystallographic directions.

SUPPLEMENTARY NOTE 4 - ESTIMATION OF SHIBA ENERGIES

The eigenvalues, ϵ_μ, of Supplementary Eq. (5) are quite involved to compute. We can project Eqs. (10) and (11) on $|\psi_\mu\rangle$ and obtain a set of equations for ϵ_μ. The local approximation [16], is to assume that $\psi_\mu(\boldsymbol{k})$ is a constant. Replacing then the sum over the first Brillouin zone by an averaged over the Fermi surface, we obtain that

$$\epsilon_\mu = \Delta \frac{1 - \alpha_\mu^2 + \beta_\mu^2}{\sqrt{1 - \alpha_\mu^2 + \beta_\mu^2 \; + \; 4\alpha_\mu^2}}, \tag{17}$$

with two solutions per channel: $\epsilon_\mu = \pm\epsilon$. From Eqs. 3, we obtain that α_μ and β_μ are given by:

$$\alpha_\mu = -\frac{\Gamma_\mu U S}{E_\mu(E_\mu + U)}, \tag{18}$$

where E_μ and Γ_μ are the center and widths of the Cr-Pb resonances, Fig. 3 main text. The intra-atomic Coulomb repulsion U and the total spin S are independent of the scattering channel. For β_μ we have

$$\beta_\mu = -\frac{\Gamma_\mu(U + 2E_\mu)}{4E_\mu(E_\mu + U)}. \tag{19}$$

Our DFT values are not accurate enough to yield precise values of the Shiba energies. Moreover, the chain of approximations leading from the DFT calculations to the final result, Eq. (17), include many approximations that render any numerical calculation valuable only as an estimation to study general trends. For these reasons, we have taken educated guesses to estimate the behaviour of the Shiba energies. Note that the calculation of images is more robust because it mainly depends on the symmetries of the orbitals and of the Fermi surfaces, that are correctly taken into account by DFT.

Our PDOS calculation, Fig. 3 main text, permit us to infer directly E_μ and Γ_μ and that the U_μ reaches large values. We analyse how the Shiba energies depend on the resonance parameters of the Cr-Pb manifold. From the PDOS (Fig.

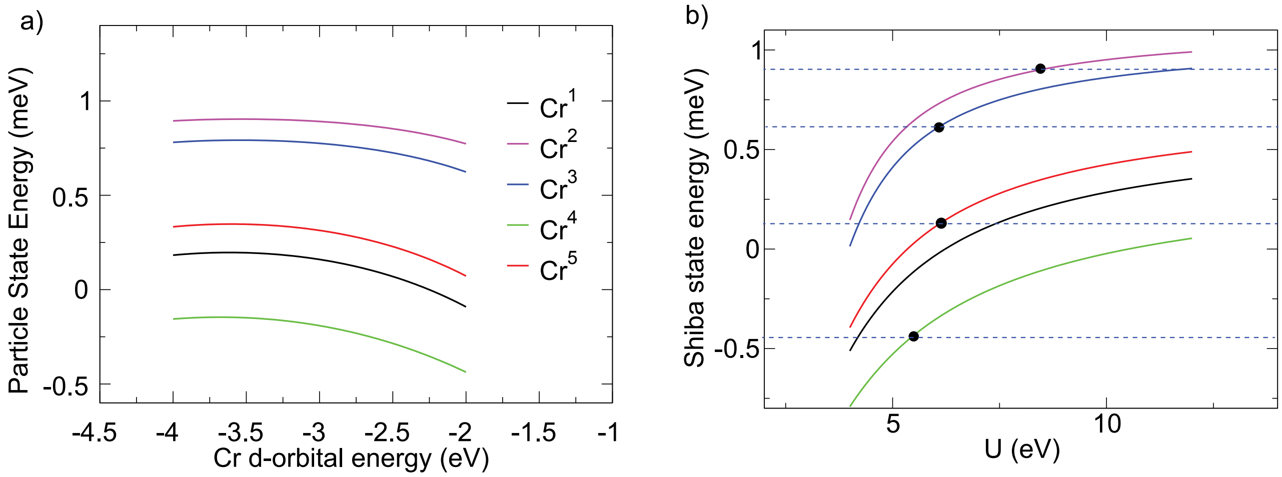

Supplementary Figure 3: Shiba particle state energies originating from the Cr five d orbitals. Energies for the five Shiba particle states, estimated from Eq. (17), as a function of (a) the Cr orbital energy E_μ and (b) intra-orbital Coulomb energy U_μ. The labels and the colours point to the same state as in Fig. 3 of the main text. Lines and dots correspond to the experimental values of the Shiba states. The negative energy corresponding to the third state, Cr^4, signals the presence of a quantum phase transition. The state Cr^1 lies far from its experimental assignment (but in agreement with its larger width in the PDOS).

3) we estimate that E_μ equal to -2.72 eV for Cr^1, -2.49 eV for Cr^2, -2.47 eV for Cr^3, -2.58 eV for Cr^4 and -2.69 eV for Cr^5. To estimate the level width from the PDOS, we removed first the large numerical broadening included for representation in Fig. 3, and obtain $\Gamma_\mu = 0.6$ eV, 0.33 eV, 0.37 eV, 0.8 eV, 0.54 eV, respectively. For both quantities, the error bar is very large, particularly because they are obtained from Kohn-Sham energy values that correspond to an effective mapping of the DFT equations and should not be considered as realistic electron energies.

In Supplementary Fig. 3a, we plot ϵ_μ for each of the 5 levels as a function of E_μ (assuming U_μ=7.5 eV). For the chosen values of U_μ and with the Γ_μ values extracted from the PDOS, we retrieve the qualitative experimental findings, namely the Shiba level ordering form most states. Specially, the simulations reproduce the negative value of the Shiba energy for the Cr^4 level, which indicates that this Shiba state has been driven beyond the quantum phase transition. The figure shows that the energies of Shiba states are not very sensitive to E_μ, but depend strongly on Γ_μ. We note that level widths can be overvalued by DFT when applied to the calculation of effective Kondo exchange couplings [7]. The values of ϵ_μ also depend strongly on U_μ (Supplementary Fig. 3b). Unfortunately, the U_μ cannot not extracted from our DFT results because the empty orbital components lie above the vacuum level.

We have used here the classical-spin approximation, make use of the fact that an exchange interaction cannot lead to spin flips inside the superconducting gap, and that spin-flips do not contribute to the interaction, Eq. (2). However, this approximation will break when the exchange interaction is so large that it can spin-flip states outside the gap. This limit can coincide with the quantum-phase transition limit in that the coupling becomes very large. However, large spin as the one in Cr, leads to low Kondo temperatures, reducing the importance of spin flips in front of the above quantum phase transition. In fact, no Kondo-like background has been observed in our experimental spectra.

SUPPLEMENTARY NOTE 5 - DECONVOLUTION OF THE TUNNELLING SPECTRA

We employed superconducting tips to increase the energy resolution of the tunnelling spectroscopy. For that reason, a double quasi-particle gap (4Δ) is observed instead of one of width 2Δ in the tunnelling spectra. In order to deconvolute the tunnelling spectra as if a normal-metal tip were employed, we used the following methodology [14].

We assume that the current is proportional to the convolution of the tip's and sample's densities of states, $\rho_T(\omega)$ and $\rho_S(\omega)$, respectively. Hence, the differential conductance is given by

$$\frac{\partial I}{\partial V} \propto \int \rho_S(\omega)\frac{\partial \rho_T(\omega - eV)}{\partial V}[f(\omega - eV, T) - f(\omega, T)]d\omega + \int \rho_S(\omega)\rho_T(\omega - eV)\frac{\partial f(\omega - eV, T)}{\partial V}d\omega \qquad (20)$$

where the Fermi distribution function is given by $f(\omega, T)$.

For the superconducting tip, we assume a BCS density of states (DOS) with a phenomenological broadening parameter γ which broadens the sharp features at the gap edges [4]:

$$\rho_T(\omega) \propto \mathrm{sgn}(\omega)\mathrm{Re}\left(\frac{\omega}{\sqrt{\omega^2 + 2i\omega\gamma - \Delta^2}}\right). \tag{21}$$

We estimate the values of Δ and γ through simulation of an experimental spectrum on a bare region of the Pb(111) surface, i.e. away from Cr atoms. We substitute Eq. (21) for both ρ_S and ρ_T in Eq. (20) and find the parameters which show the best agreement with the experimental empty tunnelling spectra. Good agreement is found for $\Delta = 1.34$ meV and $\gamma = 0.0303$ meV at $T = 1.1$ K. With the approximate ρ_T we can write down Eq. (20) in discrete form as a matrix operation on the (unknown) sample DOS:

$$\left[\frac{\partial I}{\partial V}\right] \propto \mathbf{C}[\rho_S]. \tag{22}$$

where the left hand side is a column vector with the (known) experimental spectrum, and $[\rho_S]$ is a column vector with the (unknown) DOS of the sample. By comparing Eqs. (20) and (22), and transforming the integral to a discrete sum over a finite energy range we find the elements of the convolution matrix:

$$\mathbf{C}_{ij} = (\frac{\partial \rho_T(\omega_j - eV_i)}{\partial V}(f(\omega_j - eV_i, T) - f(\omega_j, T)) + \rho_T(\omega_j - eV_i)\frac{\partial f(\omega_j - eV_i, T)}{\partial V}) \times \delta\omega, \tag{23}$$

in which ω_j are the discrete energy values and V_i the bias voltage values of the tunnelling spectrum. In general, $\mathbf{C}$ has no inverse, meaning that we cannot solve for ρ_S in Eq. (21). However, an approximate solution can be obtained by multiplying the left hand side of Eq. (22) by the Moore-Penrose pseudoinverse of $\mathbf{C}$. This procedure naturally yields the best approximate (least-squares) solution:

$$[\rho_S] \sim (\mathbf{C}^*\mathbf{C})^{-1}\mathbf{C}^*\left[\frac{\partial I}{\partial V}\right]. \tag{24}$$

In order to verify the accuracy of this procedure we re-convolute the obtained ρ_S with ρ_T through Eq. (22) and find excellent agreement with the original spectrum.

The re-convolution procedure also shows that the spectral broadening γ is crucial to resolve five peaks in the experimental spectra. Re-convolution using γ=100μV results in only three (broader) Shiba peaks, resulting in spectra similar to the results of ref. [6].

SUPPLEMENTARY NOTE 6 - AVERAGED TUNNELLING SPECTRA

As we mention in the main text, due to the small potential scattering term at the impurity, a large degree of particle-hole symmetry in the weight and amplitude of the Shiba states is expected. But spectra shows usually very different peak intensities for each component of a Shiba pair, what is probably due to the inhomogeneous shapes of the states. To prove this, we summed over all the 52×52 point spectra obtained over the 2.2×2.2-nm^2 area of Figure 2. The resulting spectrum, Fig. 4, clearly show that the surface-averaged intensities recover in part peaks with similar amplitude in their particle and hole components and, thus, the source of asymmetry in a single spectrum is partly due to the different the spatial distribution of each pair of functions $\{u_\uparrow(\boldsymbol{r}),\, v_\downarrow(\boldsymbol{r})\}$. The deconvoluted spectra give five Shiba states at energies ϵ_1=1.1, ϵ_2=0.9, ϵ_3=0.62, ϵ_4=0.45 and ϵ_5=0.125 meV in good agreement with the energies obtained from the spectra at a single spatial point.

SUPPLEMENTARY NOTE 7 - CONDUCTANCE MAP OF TWO NEIGHBOUR Cr IMPURITIES

Supplementary Figure 5 depicts the conductance maps at negative Shiba quasiparticle states, performed as described in Figure 2 for the single impurity case: a matrix of dI/dV spectra is acquired with stabilization voltage -4 mV and current 0.4 nA. The two Cr atoms are separated by four lattice unit cells ($\sim$ 1.4 nm). The spatial distributions of the Shiba states is similar to the isolated impurities, with some faint distortion in the shape of states ϵ_3^h and ϵ_4^h, but rotated by 120°, in agreement with the three possible orientation of the adsorption site respect to the Pb(111) crystal directions. The bias at which these states are found agree within 0.05 mV with the states of isolated impurities.

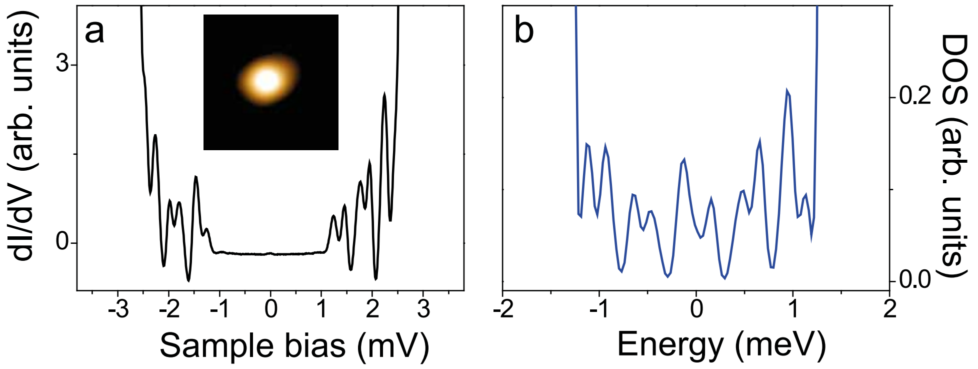

Supplementary Figure 4: The averaged spectra over the Cr adatom on Pb(111) surface (*a*) Surface integrated tunnelling spectra obtained by summing all spectra measured over the Cr atom shown in the inset (2.2×2.2 nm^2), and which used in Fig. 2d of the main text. (*b*) Deconvoluted tunnelling spectra showing the five quasiparticle bound states. The peak intensity symmetry is partially restored.

The similarities, both energetically and spatially, with the conductance maps of a single impurity in Fig. 2 lead us to the conclusion that impurities at a distance of 1.4 nm exert no influence onto each other. Furthermore, these data show that mapping Shiba states offers the possibility of investigating channel specific magnetic coupling.

---

[1] A. V. Balatsky, I. Vekhter, and J.-X. Zhu. Impurity-induced states in conventional and unconventional superconductors. *Rev. Mod. Phys.*, 78:373–433, May 2006. doi: 10.1103/RevModPhys.78.373.
[2] P. E. Blöchl. Projector augmented-wave method. *Physical Review B*, 50(24):17953, 1994. doi: 10.1103/PhysRevB.50.17953.
[3] S. L. Dudarev, G. A. Botton, S. Y. Savrasov, C. J. Humphreys, and A. P. Sutton. Electron-energy-loss spectra and the structural stability of nickel oxide:an LSDA+u study. *Physical Review B*, 57(3):1505, 1998. doi: 10.1103/PhysRevB.57.1505.
[4] R. C. Dynes, V. Narayanamurti, and J. P. Garno. Direct measurement of quasiparticle-lifetime broadening in a strong-coupled superconductor. *Phys. Rev. Lett.*, 41:1509–1512, Nov 1978.
[5] K. Fujita, I. Grigorenko, J. Lee, W. Wang, J. X. Zhu, J. C. Davis, H. Eisaki, S. Uchida, and A. V. Balatsky. Bogoliubov angle and visualization of particle-hole mixture in superconductors. *Phys. Rev. B*, 78:054510, Aug 2008. doi: 10.1103/PhysRevB.78.054510.
[6] S.-H. Ji, T. Zhang, Y.-S. Fu, X. Chen, X.-C. Ma, J. Li, W.-H. Duan, J.-F. Jia, and Q.-K. Xue. High-resolution scanning

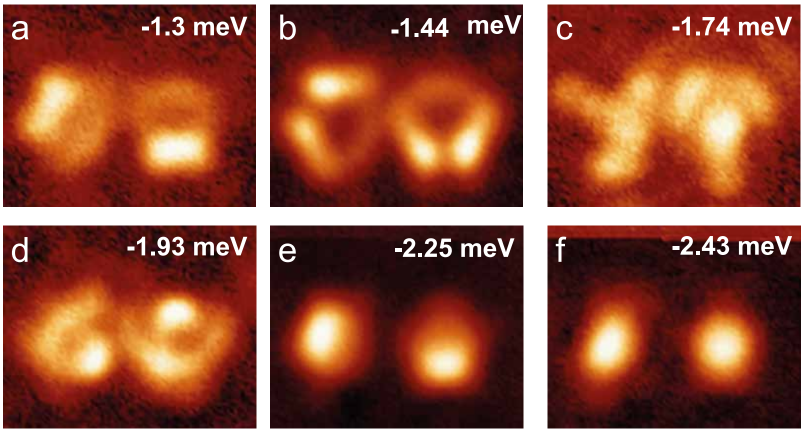

Supplementary Figure 5: Two Cr adatoms on the Pb(111) surface. Conductance images over two Cr impurities separated by 1.4 nm mapping (b-f) the amplitude of negative Shiba states ϵ_n^h and (a) the thermal replica V_6^h. The maps are construted from a matrix of spectra over the shown area, as described in the main text.

tunneling spectroscopy of magnetic impurity induced bound states in the superconducting gap of pb thin films. *Phys. Rev. Lett.*, 100:226801, Jun 2008. doi: 10.1103/PhysRevLett.100.226801.

[7] R. Korytar and N. Lorente. Multi-orbital non-crossing approximation from maximally localized wannier functions: the kondo signature of copper phthalocyanine on ag(100). *Journal of Physics: Condensed Matter*, 23(35):355009, 2011.

[8] R. Korytar and N. Lorente. Multi-orbital non-crossing approximation from maximally localized wannier functions: the kondo signature of copper phthalocyanine on ag(100). *Journal of Physics: Condensed Matter*, 23(35):355009, 2011.

[9] G. Kresse and J. Furthmüller. Efficiency of ab-initio total energy calculations for metals and semiconductors using a plane-wave basis set. *Computational Materials Science*, 6(1):15–50, 1996. doi: 10.1016/0927-0256(96)00008-0.

[10] G. C. Ménard, S. Guissart, C. Brun, S. Pons, V. S. Stolyarov, F. Debontridder, M. V. Leclerc, E. Janod, L. Cario, D. Roditchev, P. Simon, and T. Cren. Coherent long-range magnetic bound states in a superconductor. *Nature Physics*, 11:1013–1016, 2015. doi: http://dx.doi.org/10.1038/nphys3508.

[11] C. P. Moca, E. Demler, B. Jankó, and G. Zaránd. Spin-resolved spectra of shiba multiplets from mn impurities in mgb_2. *Phys. Rev. B*, 77:174516, May 2008. doi: 10.1103/PhysRevB.77.174516.

[12] M. Paulsson and M. Brandbyge. Transmission eigenchannels from nonequilibrium green's functions. *Phys. Rev. B*, 76: 115117, Sep 2007.

[13] J. P. Perdew, K. Burke, and M. Ernzerhof. Generalized gradient approximation made simple. *Physical Review Letters*, 77 (18):3865, 1996. doi: 10.1103/PhysRevLett.77.3865.

[14] J.-D. Pillet, C. H. L. Quay, P. Morfin, C. Bena, A. Levy Yeyati, and P. Joyez. Andreev bound states in supercurrent-carrying carbon nanotubes revealed. *Nature Physics*, 6:965–969, 2010. doi: doi:10.1038/nphys1811.

[15] M. Ruby, Y. Peng, F. von Oppen, B. W. Heinrich, and K. J. Franke. Orbital Picture of Yu-Shiba-Rusinov Multiplets. *Phys. Rev. Lett.*, 117(18):186801, 2016.

[16] A. I. Rusinov. Superconcductivity near a Paramagnetic Impurity. *Soviet Journal of Experimental and Theoretical Physics*, 9:85, 1969.

[17] H. Shiba. Classical spins in superconductors. *Progress of Theoretical Physics*, 40(3):435–451, 1968. doi: 10.1143/PTP.40. 435.

[18] L. Yu. Bound state in superconductors with paramagnetic impurities. *Acta Physica Sinica*, 21(1):75, 1965. doi: 10.7498/ aps.21.75.
